# Novel strategies to prevent the development of multidrug resistance (MDR) in cancer

**DOI:** 10.18632/oncotarget.19187

**Published:** 2017-07-12

**Authors:** Jinglu Wang, Nicole Seebacher, Huirong Shi, Quancheng Kan, Zhenfeng Duan

**Affiliations:** ^1^ Department of Gynecologic Oncology, The First Affiliated Hospital of Zhengzhou University, Zhengzhou, Henan 450052, People's Republic of China; ^2^ Sarcoma Biology Laboratory, Center for Sarcoma and Connective Tissue Oncology, Massachusetts General Hospital and Harvard Medical School, Boston, MA 02114, USA

**Keywords:** drug resistance, MDR, prevention, Pgp, Pgp inhibitor

## Abstract

The development of multidrug resistance (MDR) is one of the major challenges to the success of traditional chemotherapy treatment in cancer patients. Most studies to date have focused on strategies to reverse MDR following its development. However, agents utilizing this approach have proven to be of limited clinical use, failing to demonstrate an improvement in therapeutic efficacy with almost no significant survival benefits observed in cancer clinical trials. An alternative approach that has been applied is to prevent or delay MDR prior or early in its development. Recent investigations have shown that preventing the emergence of MDR at the onset of chemotherapy treatment, rather than reversing MDR once it has developed, may assist in overcoming drug resistance. In this review, we focus on a number of novel strategies used by small-molecule inhibitors to prevent the development of MDR. These agents hold great promise for prolonging the efficacy of chemotherapy treatment and improving the clinical outcomes of patients with cancers that are susceptible to MDR development.

## INTRODUCTION

**Figure 1:**
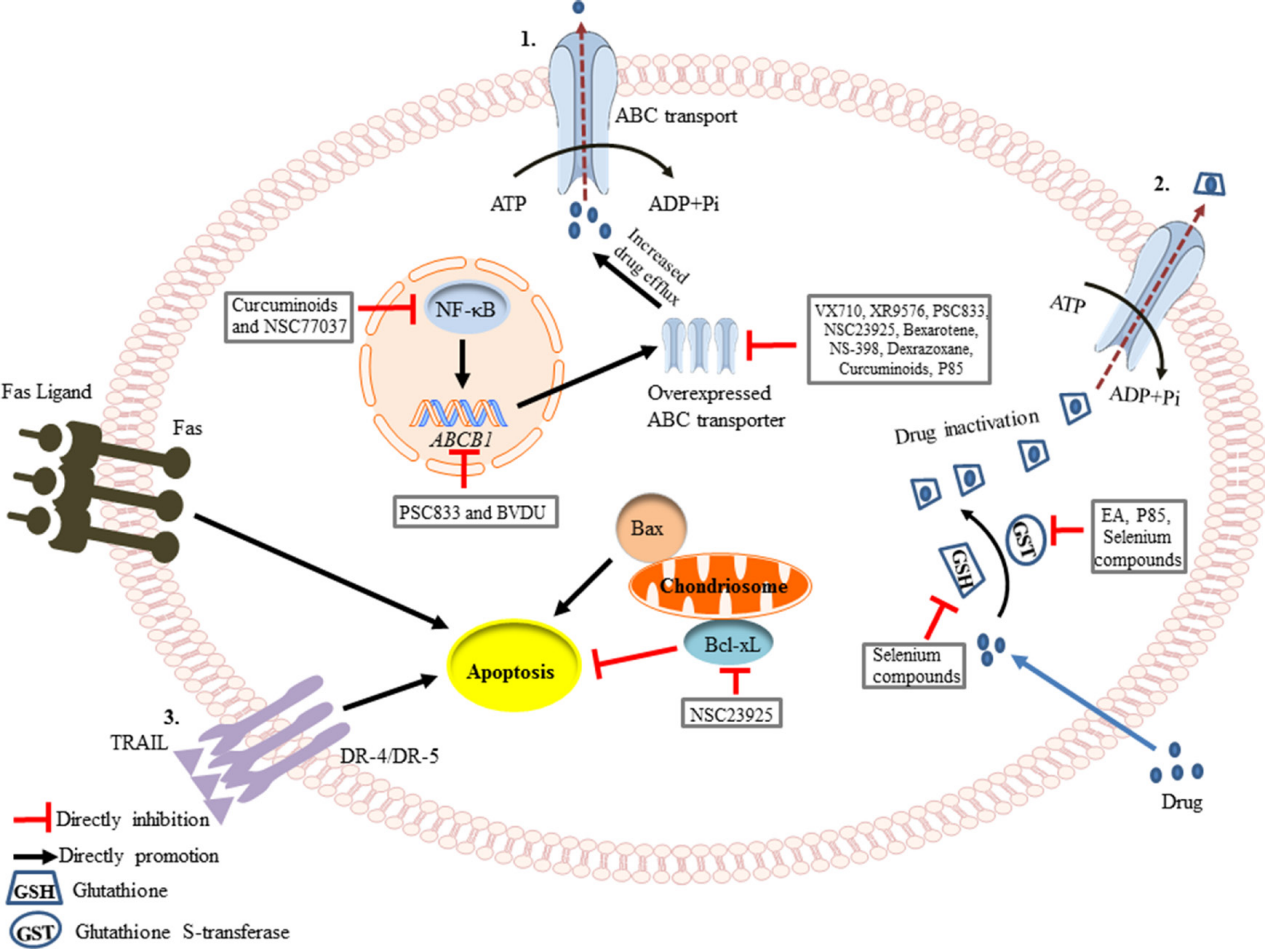
Schematic of the mechanisms involved in the prevention of drug resistance (**1**) Drug efflux is increased by the overexpression of ABC transporters during chemotherapy. Curcuminoids and NSC77037 can decrease NF-κB transcription [53, 97], while PSC833 and BVDU can suppress the *ABCB1* gene amplification [29, 48]. VX710, XR9576, PSC833, NSC23925, Bexarotene, NS-398, Dexrazoxane, Curcuminoid, and P85 directly inhibit the protein expression of P-glycoprotein (Pgp) [19, 27, 33, 34]. (**2**) Anticancer drugs can become conjugated to glutathione (GSH), leading to their inactivation. This is catalyzed by Glutathione S-transferase (GST), through utilization of the energy derived from ATP hydrolysis. Ethacrynic acid (EA), P85 and selenium compounds can inhibit GST activity [28, 30, 42], and selenium compounds even can decrease the level of GSH [43]. (**3**) Apoptosis can be initiated through two signaling pathways, the intrinsic pathway (mitochondrial-mediated) and the extrinsic pathway (death receptor-mediated). NSC23925 can inhibit the expression of Bcl-xL, an anti-apoptotic protein, thereby promoting apoptosis [21].

Multidrug resistance (MDR) is a phenomenon by which, after exposure to a chemotherapeutic agent, cancer cells develop resistance, and simultaneous cross-resistance, to a wide range of functionally and structurally unrelated chemotherapeutic drugs [1, 2]. Intrinsic or acquired MDR is one of the main reasons for chemotherapy failure, leading to the recurrence of malignant tumors and ultimately, patient relapse or death [3]. Various mechanisms have been attributed to MDR, such as enhanced drug efflux, increased DNA damage repair, reduced apoptosis, elevated autophagy, and/or altered drug metabolism [2, 4–6]. In order to improve the efficacy of chemotherapy, strategies to reverse MDR have been studied extensively over the past few decades. Three generations of MDR reversing agents, categorized according to their characteristics and chronology, have been developed, including verapamil, valspodar (PSC833), biricodar (VX710), tariquidar (XR9576) and laniquidar (R101933) [7]. A number of clinical trials of the above agents have been conducted in various different cancers types [8–11]. Unfortunately, almost no substantial survival benefits have been established, which has largely limited their widespread clinical application [12]. Recently, several studies have demonstrated that drug combinations can selectively kill resistant cells, while protecting normal cells [13–15]. For example, chemotherapeutic drugs that induce apoptosis in both normal and cancer cells, by the activation of caspases, can be used in combination with caspase inhibitors, which abrogate the chemotherapy-induced apoptosis. As a consequence of this, sensitive cells can be protected, while drug resistant cells undergo apoptosis [16]. This may be a result of enhanced drug pump expression in drug resistant cells, which export caspase inhibitors out of the MDR cells. However, these protective and selective effects may not be achieved if the normal cells express drug pumps, or if the drug resistant cells are deficient of these drug efflux proteins, thereby limiting the clinical application. The majority of previous research has solely focused on the reversal of MDR rather than prevention measures. Understanding the underlying molecular mechanisms in the evolution of MDR during the course of chemotherapy may aid in the design of novel strategies for overcoming MDR. In recent years, studies have shifted focus onto new strategies for the prevention of MDR emergence in cancer [17–20]. Several novel small-molecule inhibitors, including PSC833, VX710, XR9576, and NSC23925 have recently been confirmed as prophylactics that can prevent the induction of MDR [19, 21, 22] (Figure 1). Some of these have been applied in various clinic trails for the treatment of multiple cancer types, such as ovarian cancer, lung cancer, and multiple myeloma [9, 10, 23–25]. In this review, we summarize different model systems that prevent the development of MDR in cancer, and describe several potential mechanisms of specific agents to block drug resistance in cancers.

### Strategies to establish model systems for preventing the development of MDR *in vitro* and *in vivo*

In order to investigate strategies for the prevention of MDR in human cancer, it is necessary to establish a model of the process in which resistance develops after exposure of sensitive tumor cells to chemotherapeutic drugs. In recent years, a variety of experimental approaches have been applied to set up drug-resistant models and to evaluate the efficacy of small-molecule inhibitors on preventing the development of MDR in different cancers.

### Establishment of drug-resistant cell lines *in vitro*

**Figure 2:**
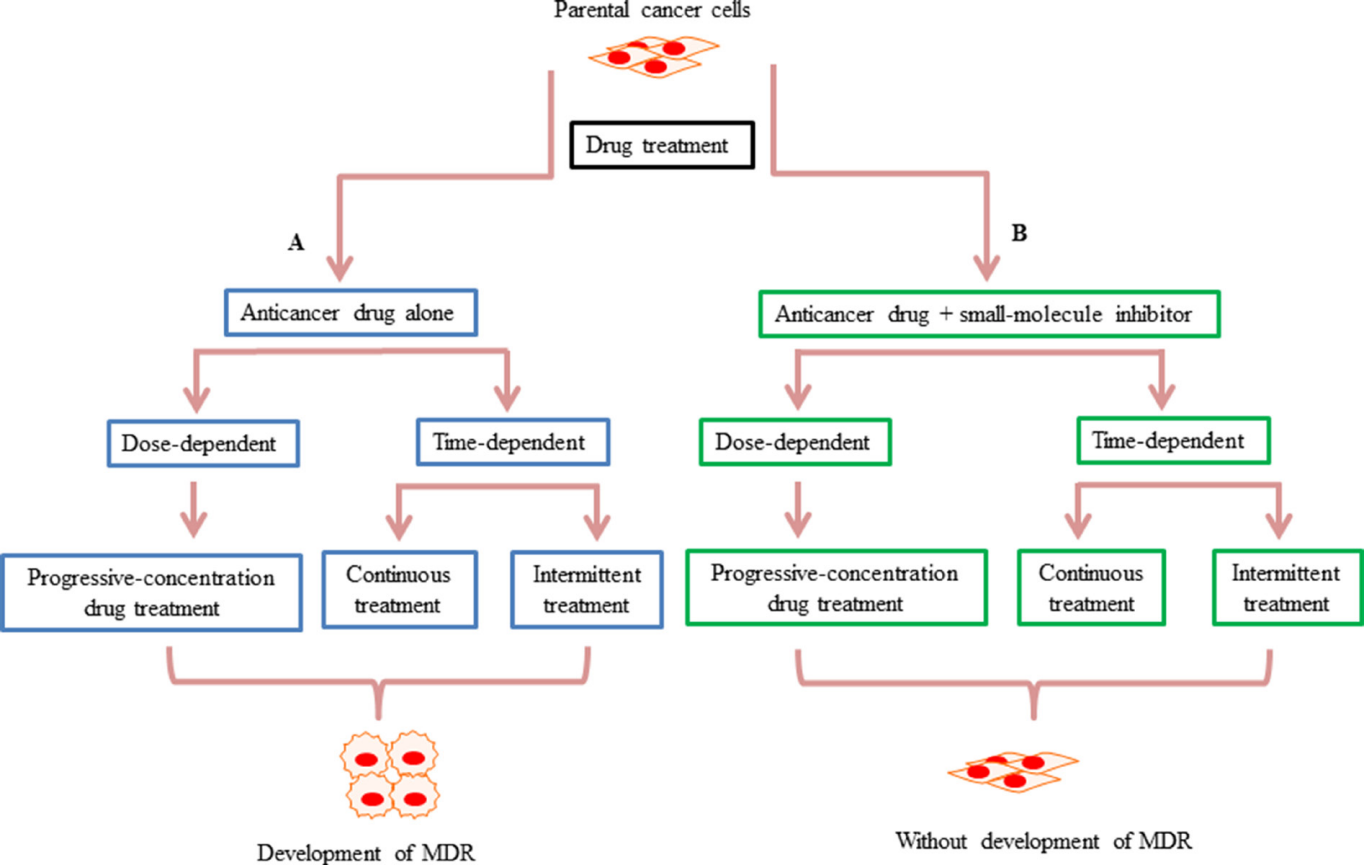
Schematic summarizing how small-molecule inhibitors may prevent the development of drug resistance *in vitro* (**A**) There are three main treatment schemes used to generate drug-resistant cell lines. In the first scheme, drug treatment is dose-dependent, whereas in the second and third the drug treatments are time-dependent. The first scheme is a progressive-concentration drug treatment, where sensitive parental cancer cells are exposed to gradually increasing concentrations of anticancer drugs [21]. The second scheme is continuous drug treatment, whereby sensitive parental cancer cells are exposed to a fixed concentration of drug for a continuous period of time [33]. In the third scheme, drug treatment is intermittent, with sensitive parental cancer cells exposed to repeated treatment cycles [34]. (**B**) In contrast to the anti-cancer agents alone causing chemotherapy resistance in these three schemes, in the presence of small-molecule inhibitors, resistance cannot develop during the drug exposure.

There are three main treatment schemes used to generate MDR cell line model systems. The first scheme requires progressive dose-dependent drug treatment, whereas the second and third schemes are time-dependent only. In the first scheme, sensitive parental cancer cells are exposed to a stepwise increase in the concentration of chemotherapeutic drug to develop MDR cell lines [19, 21, 22, 26–31]. Using this approach, cell lines with several-hundred-fold greater resistance to the chemotherapeutic drug, relative to the sensitive cell lines, can be established [21]. In the second scheme, sensitive parental cancer cells are exposed to a fixed concentration chemotherapeutic drug for a continuous and lengthy period of time [28, 32, 33]. Eventually these cancer cells will present with stable growth in the concentration of anticancer drug that would result in death of 80–90% of parental cells [33]. The third scheme requires intermittent drug treatment, i.e., sensitive parental cancer cells are exposed to repeated treatment cycles [34–37]. For example, in one study of the human breast cancer cell line, MDA-MB-231, resistance in cells was established by a 10-day treatment cycle, which included 3-days of bexarotene treatment followed by 7-days with control medium. At the end of each treatment cycle, cancer cells are harvested and re-planted into a new flask, and then re-exposed to further treatment cycles [34] (Figure 2A). In addition to studying the mechanisms of drug resistance through the comparison of sensitive and resistant cancer cells, the potential mechanisms of novel therapeutics can also be evaluated through similar experimental models. For example, the early and late stage effects of drug treatment in the development of drug resistance can be examined using chemotherapeutic drug treatment alone or in combination with small-molecule inhibitors in sensitive parental cancer cell lines (Figure 2B).

### Establishment of drug-resistant xenograft tumor models *in vivo*

Cancer cell lines *in vitro* lose many of their *in vivo* features, because they fail to address the impact of environmental signals that are present in tumors [38]. Therefore, xenograft tumor models grown in physiologically-relevant tumor microenvironments, are able to mimic the oxygen, nutrient, and hormone levels of the patient's primary tumor site, as well as maintain the genetic and epigenetic abnormalities, thereby more closely representing patient tumor growth patterns [39, 40]. As a consequence of this, responses to anticancer agents will more closely represent those seen in the patient [41].

**Figure 3:**
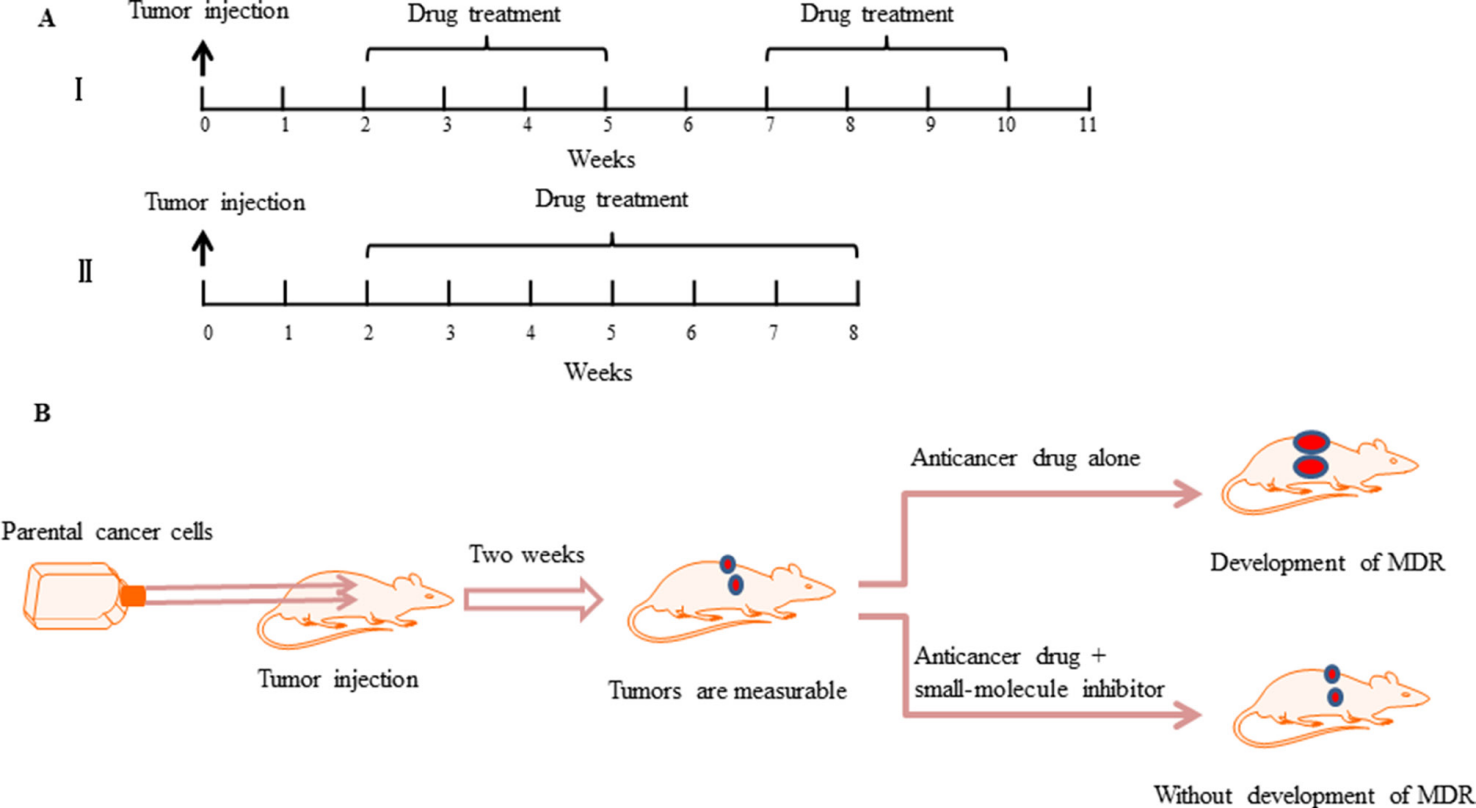
Small-molecule inhibitors prevent the development of drug resistance *in vivo* (**A**) Chemotherapy drug treatment schedule in a drug-resistant xenograft tumor model. Tumor cells are implanted subcutaneously into the left and right axial regions of 3-to-4-week-old nude mice. When the tumor volumes can be measured, two different administration methods of anticancer drugs are applied. I. The mice are exposed to a single drug concentration for 3 weeks followed by a treatment-free interval of 2 weeks. A second round of the same treatment is then continued [21]. II. The mice are exposed to a single drug concentration for six weeks, without a treatment-free interval [35]. (**B**) Schematic demonstrating that compared with anticancer drug treatments alone, combining a small-molecule inhibitor can prevent the development of drug resistance and prevent tumor growth.

In this approach, sensitive parental cancer cells in the log-phase of growth are harvested and implanted subcutaneously into right and left axial regions of 3-to-4-week-old nude mice [21]. When tumor volumes are measurable, chemotherapeutic drug treatment is commenced. There are two well established time-dependent treatment schemes. The first involves intermittent drug treatment [21, 22, 26], e.g., mice treated intraperitoneally with paclitaxel 25 mg/kg twice a week for 3 weeks followed by a treatment-free interval of 2 weeks [21]. This 5 week cycle is then continued for the duration of the experiment (Figure 3A). In contrast to this, the second scheme involves continuous drug treatment [31, 34–36, 42–45], e.g., mice can be treated intraperitoneally with paclitaxel 20 mg/kg once a week for 6 weeks [35]. The selected concentration of the cytotoxic agent is fixed at the maximum tolerated dose, which is known to cause < 10% weight loss during the experiments (Figure 3A). Moreover, the efficacy and prevention of resistance development by co-treatment of chemotherapeutic drugs with small-molecule inhibitors, both of which are initiated at the onset of first treatment, can be assessed by the measured tumor volumes (Figure 3B).

### Prevention of MDR emergence in cancer cells both *in vitro* and *in vivo*

### Pgp inhibitors

Previous studies have confirmed that MDR both in cancer cell lines and human tumor tissues is most often associated with the overexpression of the ATP-binding cassette transporter, P-glycoprotein (Pgp), also known as multidrug resistance protein 1 (MRP1), which is encoded by the *ABCB1* gene [46]. Pgp is an ATP-dependent drug-efflux pump, which exports a structurally and functionally diverse set of chemotherapy drugs from the inside of cancer cells to the outside, resulting in decreased intracellular drug accumulation [47]. While Pgp inhibitors have been shown to re-sensitize MDR cells to chemotherapeutic drugs *in vitro*, almost no survival benefits were found in clinical trials [10, 12]. This reversal strategy has thus far proven inadequate in dealing with the major clinical problem of drug resistance, and as such a new approach is needed. Perhaps, instead of reversing resistance after it has already occurred, it may be more effective to prevent or delay the occurrence of MDR with the initial chemotherapeutic drug treatment [18, 20, 48].

Several experiments have indicated that there exist a number of Pgp inhibitors that may prevent or delay the development of MDR. In parental pediatric rhabdomyosarcoma cells, which were exposed to a stepwise increase in vincristine concentration, the addition of the Pgp inhibitors, PSC833, VX710 and XR9576, prevented the development of resistance in a period of over 40 weeks [19]. In the absence of the inhibitors, vincristine resistance developed rapidly. Furthermore, all resistant cell lines expressed strikingly elevated *ABCB1* mRNA and Pgp protein levels compared with the parental cell lines that were treated in combination with the Pgp inhibitors [19] (Figure 1). In line with this, another study reported that doxorubicin (Dox) co-treatment with PSC 833 decreased cellular resistance to Dox by suppressing activation of the *ABCB1* gene and the emergence of the MDR phenotype in human uterine sarcoma cells [48]. However, these inhibitors are substrates of the Pgp transporter, acting mainly through competitive inhibition of Pgp-mediated drug efflux, and as such they lack specific target sites [7]. Consequently, clinical toxicities associated with their use at the required concentrations to inhibit Pgp function have prohibited their widespread use. For example, PSC833 and VX710 have shown unexpected pharmacokinetic interactions with paclitaxel, the major adverse effects including myelosuppression and non-hematologic toxicities [1, 49]. Therefore, the development of more potent and specific drugs that act to prevent the development of MDR is required for cancer patient treatment.

### NSC23925

NSC23925 (2-(4-methoxyphenyl)-4-quinolinyl) (2-piper-idinyl) methanol) is a long-acting methoxyphenyl piperidinyl compound, which has been identified as a noncompetitive, selective and effective inhibitor of Pgp [50]. When the effect of NSC23925 at reverse drug resistance in MDR ovarian cancer cell lines was examined, the potency of NSC23925 was been shown to be 20 and 50 fold greater than that of verapamil and Cyclosporin A (CsA), respectively [50]. Our previous studies have demonstrated that NSC23925 could prevent the introduction of paclitaxel resistance in ovarian cancer and osteosarcoma both *in vitro* and *in vivo* [21, 22, 26]. Ovarian cancer cells were cultured with stepwise increased concentration of paclitaxel in combination with or without NSC23925 [22]. After over half a year of drug treatment, cells that were exposed to paclitaxel alone grew stably in culture medium in the presence of 0.3 μM paclitaxel, without showing any signs toxicity [22]. However, cells selected with paclitaxel in combination with NSC23925 could not survive in the medium with more than 0.001 μM paclitaxel. Pgp was markedly overexpressed in all resistant cell lines, but was not expressed at detectible levels in combination treated cells. These observations indicated that NSC23925 prevented the emergence of paclitaxel resistance through specifically inhibiting the expression of Pgp, which was further confirmed in xenograft mouse models. The combination treatment remarkably prevented tumor growth compared with control saline treatment and paclitaxel treatment alone [22]. Besides the change in expression of Pgp, several apoptotic-related proteins were also detected to have changed expression after treatment in mouse tumor tissues. Anti-apoptotic proteins, such as survivin, Bcl-xL and MCL-1, showed significantly lower expression levels in the presence of NSC23925. CD44 and integrin β3, which are cancer stem cell markers, also displayed significantly decreased expression in combination-treatment tumor samples [21]. These findings suggest that co-treatment of paclitaxel with NSC23925 enhances apoptosis, which may be contributed to the prevention of paclitaxel resistance (Figure 1). The toxicity of NSC23925, determined by evaluating body weight, blood cell count and histology of organs in mouse models, showed no remarkable toxicities when exposed to NSC23925 alone or in combination with paclitaxel over the full duration of the experiment [21]. In all treatment conditions, the mice appeared to tolerate the treatments well.

### NSC77037

NSC77037, also known as Tetrandrine or CBT-1, is a bisbenzylisoquinoline alkaloid compound, which was isolated from the tuberous root of Stephania tetrandra [51]. The mechanism of NSC77037 is similar to that of NSC23925, which is to directly stimulate Pgp ATPase activity and inhibit the function of Pgp [52]. In combination with Dox, NSC77037 has been shown to prevent Dox induced MDR in human leukemia K562 cells by markedly inhibiting the *ABCB1* gene transcription [53]. K562 cells were exposed to Dox alone or in combination with three different concentrations of NSC77037. The results showed that NSC77037 could prevent Dox induced *ABCB1* expression and Pgp function in a dose-dependent manner. Several studies have shown that the inducible transcription factor, NF-κB, can upregulate *ABCB1* gene expression [54, 55]. Interestingly, while exposure of K562 cells to Dox alone elevated the expression of NF-κB in the nucleus and nuclear localization of the active protein, the Dox-NSC77037 combination decreased total and nuclear NF-κB expression as well as attenuated its DNA-binding activity, through which it inhibited the overexpression of the downstream gene *ABCB1* [53] (Figure 1).

Recently, NSC77037 was examined in a phase I clinical trial in combination with Dox to treat patients with advanced cancer [56]. Compared to other Pgp inhibitors, NSC77037 did not significantly alter the pharmacokinetics of Dox and the side effects including moderate nausea and occasional vomiting were mild. The antitumor efficacy was encouraging in the study, 25 of 34 patients were evaluable for response and 5 patients demonstrated tumor shrinkage [56]. It is currently being assessed in Phase II clinical trials of the NSC77037 in combination with paclitaxel for the treatment of solid tumors [57]. NSC77037, with lack of toxicity, could inhibit Pgp-mediated efflux of rhodamine 123 from peripheral blood mononuclear cells and normal liver to a degree, which was achieved by using other Pgp inhibitors [57].

### Dexrazoxane

Dexrazoxane, is a bis-dioxopiperazine compound, which is hydrolyzed to form a chelating agent analogous to ethylenediaminetetraacetic acid (EDTA) [58]. Many preclinical studies have shown that dexrazoxane significantly protects against Dox-induced cardiotoxicity, a major drawback of Dox [59, 60]. Moreover, clinical observation of advanced breast cancer patients treated with the FAC scheme (5-fluorouracil, Dox and cyclophosphamide) plus dexrazoxane, has shown increased overall median survival rates [61]. In contrast to Pgp inhibitors, dexrazoxane has not been described as a modulator of MDR, because it could not reverse MDR once the resistance has been formed [27]. But dexrazoxane has been shown to significantly delay the emergence of drug resistance in the human leukemia cell line K562 [27]. K562 cells that were exposed to gradually increasing Dox concentrations for 7 months in the presence of dexrazoxane, showed a marked reduction in the development of Dox resistance, compared with cells treated with Dox alone. This was reported to be a result of dexrazoxane-mediated suppression of the Dox induced upregulation of the *ABCB1* gene and Pgp protein [27] (Figure 1).

### Bexarotene

Bexarotene, also known as LGD1069 or Targretin, is a selective retinoid X receptor ligand, which has been used as an efficacious chemopreventive and chemotherapeutic agent in preclinical mice breast cancer models [62, 63]. The primary side effects of bexarotene include hypertriglyceridemia and hypothyroidism, which can be reversed or managed with corresponding drugs [64]. Bexarotene has shown great potential for chemotherapy-based combination because of its non-overlapping side effect profile with the majority of cytotoxic agents and lack of toxicity. Combination with bexarotene can prevent and reverse acquired drug resistance in several cancer cell lines both *in vitro* and *in vivo*, including advanced prostate cancer, breast cancer, and non-small cell lung cancer (NSCLC) [34–36]. For example, when the breast cancer cells, MDA-MB-231, were exposed to paclitaxel alone they demonstrated a sigmoidal concentration-dependent growth inhibition, whereas the combination of paclitaxel and bexarotene showed limited growth inhibition during the experiment [34]. Repeated exposure of cells to paclitaxel alone resulted in the development of acquired MDR. In parallel, co-treatement with paclitaxel/bexarotene delayed the growth of cells for an additional 2 months and the cells remained chemosensitive towards all the tested anticancer drugs. Similar to paclitaxel, the combination of bexarotene with other chemotherapeutic drugs, such as Dox and cisplatin, also has been shown to prevent and delay the development of MDR [34, 36]. There are several mechanisms of bexarotene that have been attributed to its preventive MDR effect [34–36]. Firstly, while resistant cells express increased levels of *ABCB1* and Pgp, they are almost undetectable in the parental cancer cells and in the co-treated cells (Figure 1). Secondly, compared with anticancer drug treatment alone, bexarotene in combination markedly suppressed the invasiveness and angiogenic potential of cancer cells. Thirdly, bexarotene maintained the genomic integrity of tumor cells by interfering with the acquisition of spontaneous mutations. These *in vitro* findings were further confirmed in mice xenograft tumor models [34–36]. In combination with a cytotoxic agent, bexarotene has been shown to dramatically decrease tumor growth compared with the cytotoxic agent alone. More recently, a study demonstrated that bexarotene could prevent and reverse gemcitabine resistance in NSCLC cells by modulating ribonucleotide reductase M1 (*RRM1*) gene amplification both *in vitro* and *in vivo* [37]. The *RRM1* gene is located in the chr11p15.5 locus, which highly expressed in gemcitabine resisitant cells, as determined by microarray gene analysis. Therefore, *RRM1* might be supressed by cell exposure to bexarotene. A phase I/II clinical trial has shown that bexarotene in combination with cisplatin or vinorelbine can prolong the survival of advanced NSCLC patients [65].

### Ethacrynic acid

Ethacrynic acid (EA) is a FDA-approved specific inhibitor of glutathione S-transferase (GST), which has been shown to enhance the cytotoxicity of anticancer drugs and reverse drug resistance [66–68]. Overexpression of GST is often regarded as a contributor to the development of cytotoxic agent resistance [67]. In a study of ovarian cancer cells that developed resistance following either exposure to low dose concentration of melphalan for 7 days, or incrementally increased dosing of melphalan for over a year, these resistant cell lines showed elevated levels of GST activity and mRNA compared with their parental cells [28] (Figure 1). However, the 7-day melphalan-resistant cells quickly reverted back to a drug sensitive phenotype in the absence of treatment within 2 weeks, indicating that the resistance was not a stable characteristic. In contrast to this, the one-year treated cell line maintained a permanent resistant phenotype, even when melphalan treatment was withdrawn. These results provide a possible explanation for enhanced efficacy of intermittent chemotherapy treatment in contrast to continuous drug delivery in clinic [69]. In addition, the development of melphalan resistance could be prevented by co-incubation with EA during the 7-day melphalan treatment in ovarian cancer cells. Moreover, this suppression of melphalan-induced resistance by EA developed in a dose-dependent manner through a reduction in GST gene expression [28].

### Selenium compounds

Selenium is an essential dietary trace element which has been extensively studied for its anticarcinogenic activity [70]. Selenium compounds, including selenite and selenomethionine, are capable of chemopreventive effectiveness through enhancing the efficacy of standard cytotoxic agents while reducing the side-effects of chemotherapy [71, 72]. Selenium compounds have been shown to prevent the emergence of drug resistance to melphalan, cisplatin, and carboplatin in ovarian cancer, both *in vitro* and *in vivo* [32, 42–45]. Interestingly, studies have shown that, selenium compounds can suppress the amplification of the GST gene, which is induced during the development of melphalan resistance [32, 42]. Selenium compounds are also able to inhibit levels of the antioxidant, glutathione (GSH), which is known to be induced during cisplatin treatment [43]. Multiple chemotherapeutic agents, such as the platinum class of drugs, are conjugated to GSH, leading to inactivation of these drugs [73] (Figure 1). Previous studies have demonstrated that elevated GSH level is correlated with platinum drug-induced resistance [74, 75]. Furthermore, inclusion of selenite compounds with cisplatin treatment prolonged and enhanced the efficacy of chemotherapy treatment, resulting in reduced growth of ovarian tumor xenografts [44]. Notably, when cells from platinum resistant or sensitive tumors were transplanted into new animals, the derivative tumors retained the drug resistant or sensitive phenotype of the tumors from which they originated [43–45]. These results support the hypothesis that prevention of resistance may result from a genetic or epigenetic effect of selenium compounds.

### BVDU

(E)-5-(2-Bromovinyl)-2-deoxyuridine (BVDU, RP101) is well established as a highly potent and selective anti-viral agent, inhibiting both herpes simplex virus type 1 (HSV-1) and varicella-zoster virus (VZV) [76]. BVDU has also been shown to suppress 2-amino-6-mercaptopurine-induced SV40 viral amplification in Chinese hamster cells and abolished triethylene melamine (TEM)-induced mitotic recombination in yeast [77]. Importantly, gene amplification and recombination may be regarded as contributors to acquired chemoresistance. Gene recombination is functionally related to amplification, indeed the first step of both processes are the same [78, 79]. In a study by Fahrig et al., mouse leukemia cell lines were exposed to 4 weeks of increasing concentrations of Dox in the presence or absence of BVDU [29]. In the cells treated with BVDU, Dox resistance did not develop, which was likely due to the suppression of *ABCB1* gene amplification and expression [29] (Figure 1). Similarly, BVDU has also been reported to prevent drug resistance to methotrexate (MTX), by suppressing the chemotherapy-induced amplification of the dihydrofolate reductase (*Dhfr*) gene in 3T6 cells [80]. Further studies also revealed that in combination, BVDU strengthened cytotoxicity of all tested chemotherapy agents, including DOX, mitomycin C, mitoxantrone, glufosfamide, and cisplatin, by inducing apoptosis *in vitro* and dramatically enhanced tumor regression *in vivo* [80]. While the anticancer drugs alone resulted in significant body weight loss in rats, this was partly inhibited with BVDU co-treatment. This may indicate BVDU co-treatment can reduce nonspecific toxicity and optimized antitumor efficiency. There are several possible mechanisms explaining the modulation by BVDU. Firstly, BVDU enhances the activity of NAD(P)H: quinone oxidoreductase 1 (NQO1), a FAD containing quinone reductase, which is frequently decreased in MDR cancer cells [81]. Secondly, BVDU inhibits the expression of the oncogene DDX1 and the DNA repair associated enzymes, UBE2N and APEX. Thirdly, BVDU inhibits cell survival pathways, involving STAT3 and JUN-D, leading to promotion of apoptotic pathways [82, 83]. Lastly, during recovery, BVDU promotes upregulation of microfilamental proteins and suppression of ATP generating proteins [80].

### NS-398

N-[2-(cyclohexyloxy)4-nitrophenyl]-methanesulfonamide (NS-398), is a specific inhibitor of Cyclooxygenase-2 (COX-2), an enzyme required for the formation of prostanoids [84]. Reports have shown that COX-2 is upregulated in a wide range of solid tumors, promoting angiogenesis, invasiveness and anti-apoptotic activity [85, 86]. Selected COX-2 inhibitors, such as meloxicam and rofecoxib, have been reported to reverse MDR by reducing the expression of ABC-transporter proteins [87, 88]. Similar to this, treatment with NS-398 alone has been shown to not affect cell proliferation in parental breast cancer cells, but is able to prevent or reduce the development of the chemoresistance phenotype in breast cancer cells [33]. In a study of breast cancer cells exposed to low dose Dox for 10 days or 2 months in order to develop drug resistance, demonstrated both cell lines developed enhanced levels of Pgp and COX-2 [33]. The resistance formed in the 10-day Dox treated cells was shown to be reversible following either drug withdrawal or with NS-398 co-treatment. Whereas, in the 2 month-resistance model, the “permanent” resistance that was established with Dox treatment, did not occur with NS-398 treatment, and consequently, the cytotoxic effects of Dox remained potent. The application of NS-398 in combination is believed to have prevented the development of Dox resistance, through suppression of Pgp and COX-2 activity (Figure 1). Therefore, by decreasing Pgp, significantly more intracellular drug accumulation and retention was observed *in vitro*.

### Curcuminoids

Curcuminoids, are a class of natural phenolic coloring compounds, which are isolated from the rhizomes of Curcuma longa [89]. Curcuminoids show a broad range of biological activities, including potent anti-inflammatory, anti-oxidant and anti-tumor properties [90, 91]. Increasing evidence indicates that curcuminoids act to suppress proliferation, metastasis, invasion and angiogenesis of human cancers, through interactions with several intracellular signal transduction pathways [92, 93]. Experimental results and clinical trials have demonstrated that curcumin (Cur) is non-toxic even at high concentrations [94]. Furthermore, curcuminoids have the potential to block acquired resistance induced by adriamycin (ADM), through the downregulation of *ABCB1* mRNA and Pgp protein [95]. Curcuminoids contain at least three active forms: curcumin (Cur), demethoxycurcumin (D-Cur) and bisdemethoxycurcumin (BD-Cur) [96]. The anti-chemoresistance activity of these three forms of curcumin has been compared in a study by Xu et al. and the possible mechanisms of activity explored [97]. In this study, human leukemia cells were pretreated with or without Cur, D-Cur and BD-Cur for 24 h, followed by addition of ADM, and then co-incubated for another 72 h. The results revealed that curcuminoids significantly inhibited the expression of *ABCB1* and Pgp. D-Cur was the most active drug, followed by BD-Cur, with Cur showing the least activity. Western blot analysis was also applied to explore the effect of curcuminoids on NF-κB transcriptional activity, as *ABCB1* gene expression is known to be regulated by NF-κB [55] (Figure 1). BD-Cur also proved to have the greatest activity in suppressing the nuclear translocation of NF-κB, followed by Cur and then D-Cur, which interestingly did not correlate with the trend observed for the preventive activity of the three curcuminoids [97].

### Pluronic P85

Pluronic compounds are a remarkable example of polymers, which hold tremendous potential for the advancement of drug delivery applications, due to a number of preferable characteristics, such as biocompatibility, tunable chain length and a number of other properties [98–100]. Pluronic compounds are an A-B-A amphiphilic block copolymers, containing hydrophilic poly (ethylene oxide) (PEO) blocks and hydrophobic poly (propylene oxide) (PPO) blocks [101]. In MDR cancer cells exposed to Pluronics, drug sensitivity to anticancer drugs was reinstated [102, 103]. Because of the lipid-like amphiphilic character, Pluronics can effectively move into cellular membranes and significantly inhibit drug-efflux transporter proteins, such as Pgp, leading to increased intracellular drug accumulation [104]. Pluronic compounds have been shown to induce intracellular ATP depletion, which prevents the activity of Pgp [103]. Pluronic P85 has also been demonstrated to prevent the development of MDR in the human breast carcinoma cell line, MCF7 [30]. MCF7 cells were exposed to increasing concentrations of Dox with or without P85 for 305 days *in vitro*. In the cells treated with 1000 ng/ml of Dox alone, stable growth in culture medium was eventually obtained, whereas Dox-P85 co-treated cells were unable to survive in the culture medium supplemented with more than 10 ng/ml of Dox [30]. P85 was shown to prevent Dox resistance through inhibition of *ABCB1* and Pgp expression, and it also simultaneously suppressed expression of the GST pi gene, which is also a relevant indicator of chemotherapy resistance (Figure 1). Furthermore, DNA microarray analysis revealed that co-treatment with P85 abolished alterations of genes that are involved with drug metabolism, apoptosis, stress response, transcriptional factors and tumorigenesis [30]. Therefore, application of Pluronic P85 in the clinic might potentially enhance therapeutic outcomes in patients with breast cancer. Similar effects of P85 have also been reported in leukemia cells both *in vitro* and *in vivo* [31]. The mechanisms of P85 on MDR prevention in leukemia were reportedly similar to that in breast cancer.

### Conclusion and future perspective

MDR is a major obstacle that severely limits the efficacy of clinical chemotherapy in the treatment of cancer. Experimental drug resistance cancer models have contributed to the identification of many of the underlying mechanisms involved in the development of MDR. Many attempts have been conducted in clinic trials to overcome drug resistance, but have been met with limited success. Preventing the development of drug resistance holds great value as a novel strategy for anticancer treatment [17, 19, 22, 35]. Recent studies have demonstrated that several small-molecule inhibitors, including Pgp inhibitors, are capable at preventing the development of MDR when co-treated with cytotoxic drugs in different *in vitro* and *in vivo* model systems (Table 1). Preventing or delaying the emergence of drug resistance is likely to enhance the effectiveness of chemotherapy and improve clinic outcomes for patients with cancer. This review highlights that a pilot study is necessary to optimize the concentrations required for long-term exposure to chemotherapeutic agents and small-molecule inhibitors to prevent MDR development and consequently poor patient treatment outcomes. Further studies on the mechanisms of prevention of MDR and clinical testing of these agents are needed to determine their efficacy in the treatment of human cancers.

**Table 1:** Small-molecule inhibitors prevent the development of drug resistance in human cancer

Compound	Functional mechanism	Cytotoxic agent used in combination	Cancer type	Reference
VX710	inhibits the expression of *ABCB1* mRNA and Pgp	vincristine	rhabdomyosarcoma	[19]
XR9576	inhibits the expression of *ABCB1* mRNA and Pgp	vincristine	rhabdomyosarcoma	[19]
PSC833	inhibits the expression of *ABCB1* mRNA and Pgp	vincristine	rhabdomyosarcoma	[19]
	suppresses the activation of *ABCB1* gene; inhibits the appearance of MDR phenotype	doxorubicin	uterine sarcoma	[48]
NSC23925	suppresses the expression of Pgp and enhances apoptosis	palitaxel	ovarian cancer	[21, 22]
	inhibits the expression of Pgp	palitaxel	osteosarcoma	[26]
NSC77037	inhibits the expression of *ABCB1* gene and Pgp; decreases NF-kB transcription and protein activity	doxorubicin	leukemia	[53]
Dexrazoxane	inhibits the expression of *ABCB1* mRNA and Pgp	doxorubicin	leukemia	[27]
Bexarotene	inhibits the expression of *ABCB1* mRNA and Pgp; suppresses angiogenic and invasiveness potential;	paclitaxel; doxorubicin; cisplatin	advanced prostate cancer; breast cancer; non-small cell lung cancer	[34–36]
	decreases spontaneous mutation rate suppresses the amplification of *RRM1* gene	gemcitabine	non-small cell lung cancer	[37]
Ethacrynic acid	inhibits the levels of GST activity and mRNA	melphalan	ovarian cancer	[28]
Selenium compounds	inhibit the expression of GST mRNA and enzyme activity	melphalan	ovarian cancer	[32, 42]
	inhibit the level of GSH	cisplatin	ovarian cancer	[43]
	maintain the sensitivity following transplantation	cisplatin; carboplatin	ovarian cancer	[43–45]
	prolong and enhance the efficacy of chemotherapy treatment	cisplatin	ovarian cancer	[44]
BVDU	inhibits the amplification of *ABCB1* gene	doxorubicin	leukemia; fibrosarcoma; mammary adenocarcinomas	[29, 80]
	suppresses the amplification of *Dhfr* gene enhances the activity of NQO1;	methotrexate	fibrosarcoma	[80]
	inhibits the expression of oncogenes and DNA repair enzymes; suppresses the proteins of survival pathways or ATP generation;	doxorubicin; mitomycin C; mitoxantrone	hepatosarcoma	[80]
	up-regulates microfilamental proteins			
NS-398	inhibits the expression of Pgp and COX-2	doxorubicin	breast cancer	[33]
Curcuminoids	inhibits the expression of ABCB1 mRNA and Pgp; suppress the nuclear translocation of NF-kB	adriamycin	leukemia	[95, 97]
Pluronic P85	inhibits the expression of *ABCB1* mRNA and Pgp; abolishes the alterations of genes implicated in drug resistance	doxorubicin	breast cancer; leukemia	[30, 31]
	suppresses the level of GST pi mRNA	doxorubicin	breast cancer	[30]
